# Measuring the size and growth of single cells

**DOI:** 10.52601/bpr.2022.210036

**Published:** 2022-06-30

**Authors:** An Gong, Mingwei Min

**Affiliations:** 1 Guangzhou Laboratory, Guangzhou 510030, China; 2 Guangzhou Institutes of Biomedicine and Health, Chinese Academy of Sciences, Guangzhou 510530, China

**Keywords:** Cell volume, Cell mass, Cell size, Cell density

## Abstract

The size and growth of a cell can be described by three related physical parameters: volume, density and mass. All the three are coupled to numerous biochemical reactions and biophysical properties of a cell. It is therefore not surprising that cell size and growth pattern are tightly regulated across all kingdoms of life. Indeed, deregulation of cell size and growth has been found to be associated with diseases. Yet, how cells regulate their size and how cell size connects to cell function remain poorly understood, partly due to the difficulties to precisely measure the size and growth of single cells. In this review, we summarize methods of measuring cell volume, density, and mass, and discuss how the new technologies may advance our understanding of cell size control.

## INTRODUCTION

A fundamental idea in biology is that structure determines function. While this notion is the most intuitive for multi-cellular organisms, it is also true for a single cell. As one of the basic structural parameters of a cell, cell size directly impacts the concentration of cellular components, which dictate the rates of biochemical reactions (Schmoller and Skotheim 2015), biophysical properties such as crowding (Delarue* et al.* 2018), and organelle homeostasis (Miettinen and Björklund 2016). In humans, cell volume varies across more than five orders of magnitudes, ranging from sperm cells of about 30 µm^3^ (Bionumber database (Milo* et al.* 2009), BNID 109891, 109892) and red blood cells of about 100 µm^3^ (BNID 107600), to fat cells of 600,000 µm^3^ (BNID 107668) and egg cells of 4,000,000 µm^3^ (BNID 101664). Yet within the same tissues and the same cell types, individual cells deviate very little from their characteristic size (Ginzberg* et al.* 2015). The extensive inter-cell-type variation implicates that the size of a cell is specified to fulfill its certain functions, and the intra-cell-type uniformity indicates that the cells actively maintain their sizes and that the size homeostasis is critical for the function of a cell. The deviation from cell size control is often a signature of diseases. For example, the loss of cell size uniformity has been widely observed in malignant tumors, as one of the most distinctive morphological features that separate cancerous tissue from its healthy counterpart, and as one of the most widely used histological features in pathology diagnosis (Majno and Joris 2004). The increase of size variation is likely a manifestation of growth and proliferation dysregulation in cell size control. Despite of the clear correlation between cell size and function, it remains poorly understood how cell type and function restrain the size of a cell or vice versa, and how cells measure and maintain their size in accordance with their function. Recently, cytoplasmic dilution has been proposed to play a causal role in cellular senescence (Neurohr* et al.* 2019), a permanently non-proliferative state characterized by large and flat cell morphology (Hayflick and Moorhead 1961). It was further shown *in vivo*, that enlarging hematopoietic stem cells (HSC) induces the decline of their reconstitution potential and that preventing environmental insults- or aging-induced HSC enlargement ameliorates their fitness loss (Lengefeld* et al.* 2021) These results suggest that size can indeed determine function. But even in these rare cases where a causal relationship between cell size and cell function is evident, the detailed mechanistic underpinning, as of how cell volume and density impinge on the cell fate choices, remains obscure. The understanding of mechanisms is complicated in part by the elaborate nature of cell size related changes. It is also hindered by the technical challenge to weigh an entity as small as a cell. Comparing to the suite of molecular toolkits to measure the activity of signaling pathways that control cell growth and proliferation, the technology to measure cell size and growth *per se* is still in its infancy.

In this review, we focus on the methodological advances in measuring cell size, including cell volume, density, and mass (Table 1). These quantities are not independent since mass is the integral of density across the volume. Here, we classify methods based on the physical quantity (*i*.*e*., volume, mass or density) they directly measure. We revisit the established methods, consider the evolution of new methods, and expand upon potential future development. For conceptual progress of cell size control, we refer the readers to other excellent reviews (Ginzberg* et al.* 2015; Neurohr and Amon 2020; Schmoller 2017).

**Table 1:** A collection table of methods measuring cell volume, mass, and density

Method	Principle-physical quantity	Precision (measurement error)	Time series measurement	Throughput (cells/ experiment)	Adherent property	Reference
Coulter counting	Conductance-cell volume	NR	No	>10^4^	Suspension	Henriquez* et al.* 2004
Flow cytometry	Forward light scattering-cell size	10%–20% of the cell volume	No	>10^5^	Suspension	Tzur* et al.* 2011
3D cell imaging	Cell boundary-cell volume	NR	Yes	~100–1000 cells	Adherent	Errington and White 1999; Padovan-Merhar* et al.* 2015; Xie and Skotheim 2020
Fluorescence exclusion imaging	Fluorescent dye exclusion-cell volume	relative precision at 1% of the cell volume	Yes	>1000	Adherent	Zlotek-Zlotkiewicz* et al.* 2015
Mircofluidic resonator	Vibration frequency-cell mass	0.02–0.10 pg	Yes	1–60	Suspension	Burg* et al.* 2007; Cermak* et al.* 2016; Son* et al.* 2012
Micro-electro-mechanical systems	Vibration frequency-cell mass	8.5 pg	Yes	30	Adherent	Park* et al.* 2010
Raman imaging	Characteristic Raman peaks-concentration of lipid and protein	15 mg/mL	Yes	~100–1000 cells	Adherent	Oh* et al.* 2019
Refractive index tomography	Refractive index-cell density	1–10 mg/mL	Yes	2–400	Both	Li* et al.* 2019; Park* et al.* 2018
Fluorescent labeling	Primary amine-protein concentration	NR	No	>10^5^	Adherent	Kafri* et al.* 2013
Particle diffusion	Cell crowding state-relative cell density	NR	Yes	1	Adherent	Delarue* et al.* 2018
NR: Not reported						

## MEASUREMENT OF CELL VOLUME: COULTER COUNTER, FLOW CYTOMETRY AND MICROSCOPY

**Figure 1:**
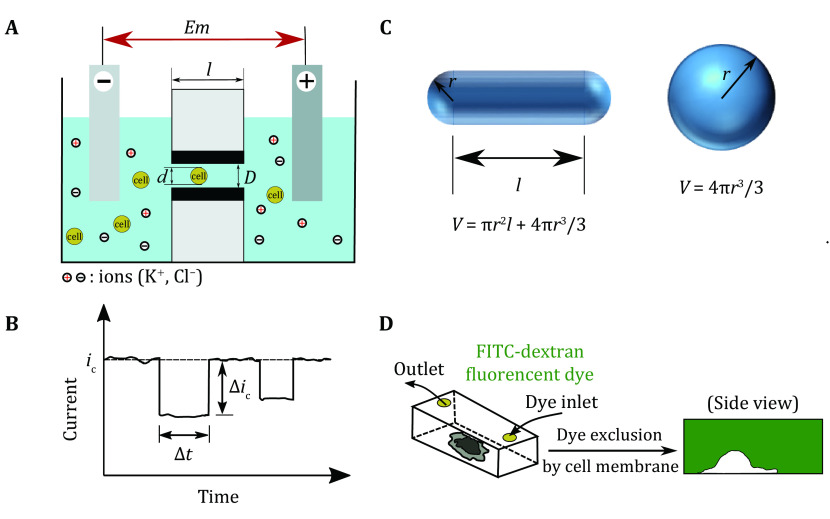
Methods to measure single-cell volume. **A** A schematic diagram showing the basic principle of a Coulter counter. A chamber containing an electrolyte solution is divided into two parts by a membrane with a single channel in it. The channel can conduct current. When a particle with an appropriate size and charge enters into the channel, the ion current reduces. **B** Representative Coulter counter data showing the current decrease Δ*i*_c_ (indicating particle size) and the transit time Δ*t* (indicating the particle charge). Panel **A** and **B** are redrawn from Henriquez* et al.* (2004). **C** The geometrical parameters of regular shape objects. **D** The principle of measuring cell volume with fluorescence exclusion method. The membrane impermeable dye FITC-dextran is mixed into the cell culture medium. The cell is negatively stained, and its volume can be calculated

Cell volume can be measured with a Coulter counter, a flow cytometer, or a light microscope. The basic principle of Coulter counting is that the conductance of a small channel changes when a cell passes through the channel (Fig. 1A and 1B). The change of conductance is determined by the length (*l*) and diameter (*D*) of the small channel and is also positively related to the cell radius (*d*) (Henriquez* et al.* 2004). The *l* and *D* are the fixed properties of the Coulter counter and cell volume can be calculated from *d*. Flow cytometry is another widely used method to measure the volume of suspended cells. In a flow cytometer, single-cell droplets pass through a set of laser light one at a time, where the light scattering and fluorescent intensity can be recorded by detectors. The intensity of forward scatter (FSC), the scattering measured along the path of the laser light, positively correlates with the volume of the cell. This optical parameter can be used for deriving cell volume with proper calibration (Tzur* et al.* 2011). The flow cytometer is easily accessible equipment for most cell biology labs, and it also has the advantage of correlating cell size with other fluorescent measurements at the level of single cells. However, its precision is limited as the FCS is also affected by the refractive index of a cell, which might vary from cell to cell. The two methods mentioned above do not allow recurrent measurement of the same individual cells. To measure adherent cells, one needs to lift the cells off the dish first. This preprocessed step limits the time resolution of the cell growth pattern measurement, and may affect the growth of adherent cells (Conlon and Raff 2003).

Microscopy techniques, on the contrary, offer a direct observation of cells in their native environment. For cells with a simple shape, such as rod (*e*.*g*., *Escherichia coli* and *Schizosaccharomyces pombe*) or sphere (*e*.*g*., lymphocytes), the cell volume can be easily derived from the cell’s geometrical parameters (Fig. 1C) (Facchetti* et al.* 2019). The irregular cell shape can be characterized with 3D scanning methods such as the con-focal microscopy, when the cell membrane or cytoplasmic components are fluorescently labeled (Fig. 1D) (Errington and White 1999; Padovan-Merhar* et al.* 2015). With the recent advance in the microscopy field, multi-photon microscopy has allowed recursive imaging of deep tissues in live animals. This is so far the only method of measuring cell size dynamics in living mice (Mesa* et al.* 2018; Xie and Skotheim 2020). Comparing to the scanning-based methods, wide-field microscopy has the advantage of faster imaging and thus higher throughput. To enable precise cell size measurement with wide-field microscopes, a fluorescence exclusion method has been developed. It involves placing cells in a fixed-height microchamber filled with cell membrane impermeable dyes, where the exclusion of fluorescent dye by cells creates a negative contrast image (Fig. 1E) (Gabella* et al.* 2014; Zlotek-Zlotkiewicz* et al.* 2015). However, active endocytosis enables cells to internalize the dye from the medium, which introduces additional variables and decreases the contrast over time. Quantitative phase imaging methods can also be used to reconstruct the cell shape. We will discuss this method in detail in the part of cell density measurement.

## WEIGHING THE MASS OF A CELL: RESONATOR-BASED METHODS

**Figure 2:**
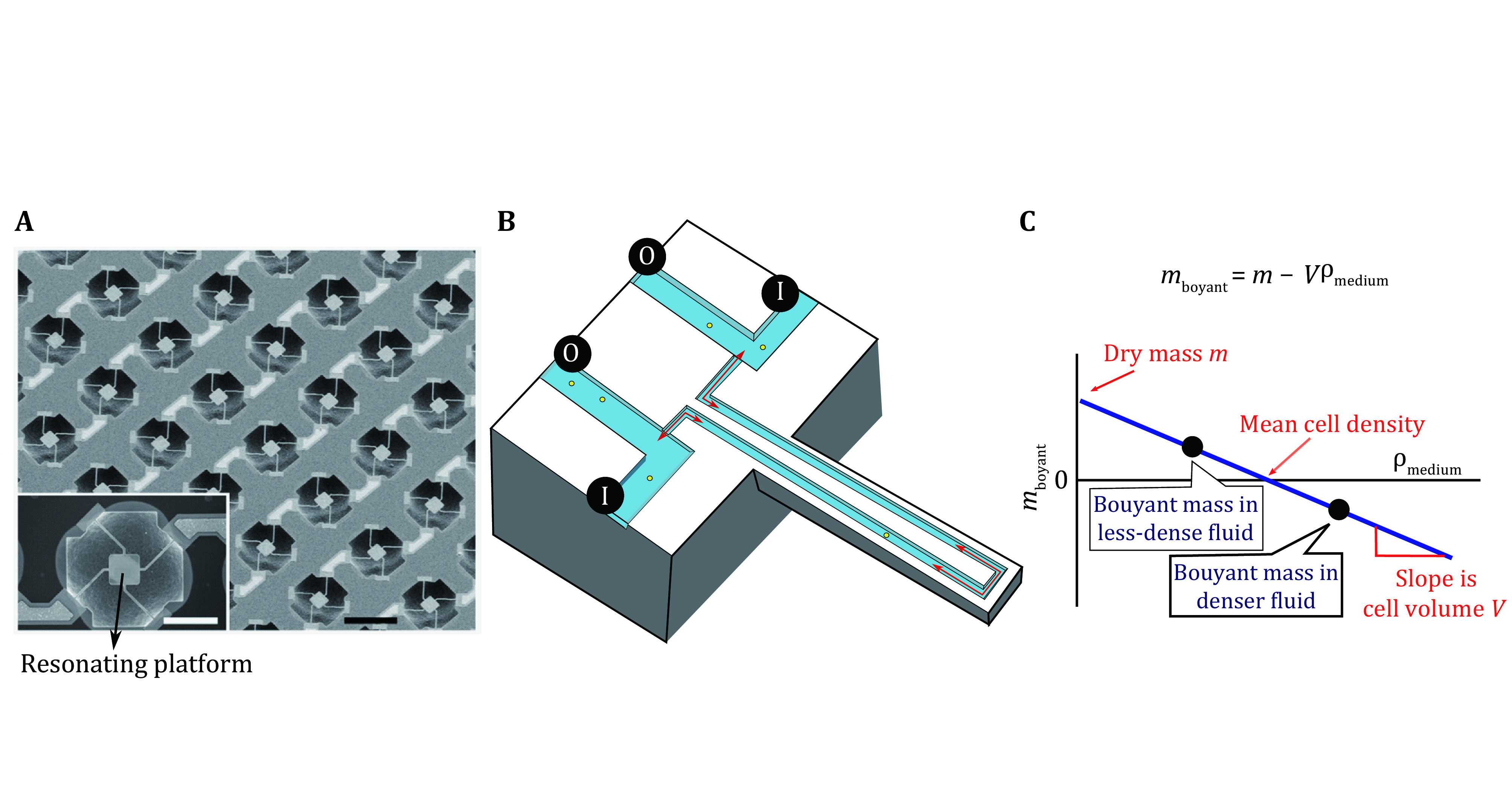
Measuring cell mass with vibration-based methods. **A** The matrix of micro-scale resonators on a micro-electro-mechanical system. The figure is adapted from Park* et al.* (2010). **B** The resonant frequency of a vibration system (a cantilever in the figure) depends on the mass of the system. The outlet (O) and inlet (I) is used for controlling the flow direction, figure is redrawn from Godin* et al.* (2010). **C** The cell volume, mass and mean density can be derived using Archimedes’ principle if the cell’s buoyant mass is measured in two fluids with different densities, figure is redrawn from Grover* et al.* (2011)

The resonant frequency of a resonator depends on its mass (Johnson and Mutharasan 2012). When a cell is placed in a vibrating system, the frequency shift of the resonator caused by the mass change can be detected (Fig. 2A). This principle has been used to design the micro-electro-mechanical systems (MEMS), where cells are attached to a resonating platform. By aligning a matrix of micro-scale resonators, the MEMS chip can measure the weight of these adherent cells at high throughputs (Fig. 2C) (Park* et al.* 2010). However, the resolution of resonator frequency is limited due to damping of the resonator by surrounding liquid, thus limits the resolution and precision of mass measurement. Also, the MEMS cannot distinguish the weight of single cells from a cell cluster or a colony and is therefore unable to track a single cell’s mass over multiple generations. A different configuration, the suspension microchannel resonator (SMR), substantially improves the resolution of resonator frequency by replacing the resonating platform in the liquid with a cantilever in a vacuum. In SMR, cells are repeatedly flowed through a microfluidic channel in a cantilever. The difference between the cell mass and the media mass displaced by the cell can be derived, named buoyant mass (Godin* et al.* 2010). The SMR is the most accurate method for measuring buoyant mass so far, with a precision of around 20 femtograms, which is approximately 0.02% of the mass of an average mammalian cell (Burg* et al.* 2007; Son* et al.* 2012). The SMR can be coupled to an imaging system where temporal tracking of mass and other signaling events (*i*.*e*., via fluorescent reporters) is possible (Kang* et al.* 2020). When measuring the buoyant mass changes of a cell in two suspension media with different densities, the volume and mass of the cell can then be derived using Archimedes’ principle (Grover* et al.* 2011). Hence the mean cell density can also be obtained (Fig. 2B). The throughput of the SMR system can be improved by using an array of connected cantilevers, to allow multiple cells flowing in a queue, one cell in one cantilever at a time (Cermak* et al.* 2016). With its temporal resolution and unparalleled precision, SMR has become the state-of-the-art method for measuring single-cell mass, optimal for suspension cells.

## MEASURING CELLULAR DENSITY

**Figure 3:**
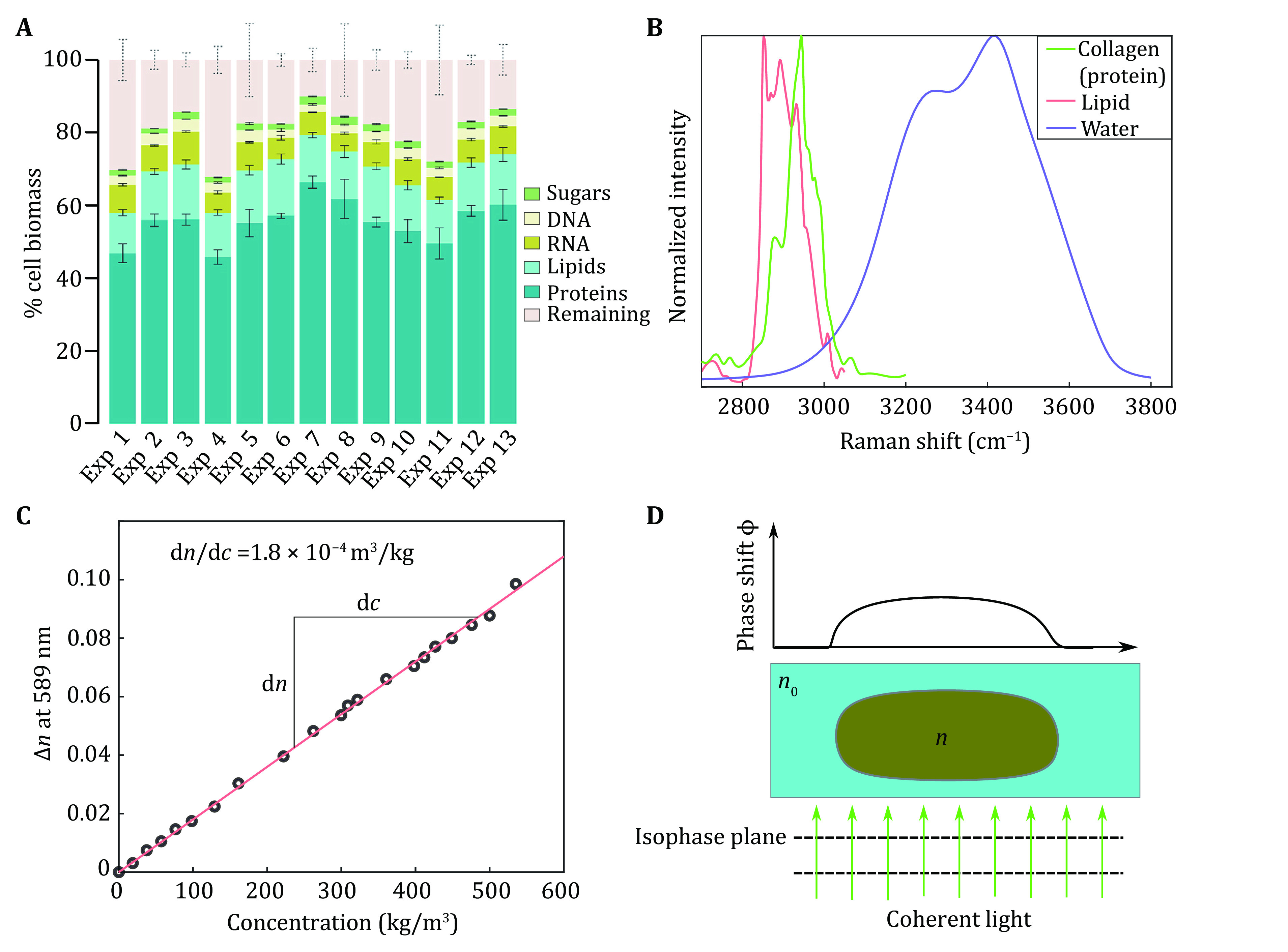
Principles used in single-cell density measurement. **A** Relative biomass composition of various Chinese hamster oocyte cell lines under defined conditions, figure is adapted from Széliová* et al.* (2020). **B** Protein (collagen from the mouse as an example) and lipid (liposome with DOPC : cholesterol = 6 : 4 as an example) have distinct peaks in the stimulated Raman scattering (SRS) intensity spectra, which can be used for quantification, figure is redrawn based on data from Faried* et al.* (2019) and Rygula* et al.* (2013). **C** The refractive index linearly correlates with protein concentrations, figure is redrawn based on the data from Barer and Tkaczyk (1954). **D** Using phase shift for single-cell density measurement: the wavefront (*i*.*e*., isophase surface, indicated as dashed lines) of a coherent light beam changes after passing through the cell with a refractive index *n*. The cell mass can be estimated by calculating the refractive index according to the linear relationship shown in Panel C

In biology, density gradient centrifugation is one of the most widely used methods to separate molecules and cells with distinct densities. Nonetheless, like many biochemical methods, this method only applies to bulk populations of cells (Allen* et al.* 2006). For single cells, cell density is usually approximated as the sum of its main components’ concentration. Typically, the mammalian cell biomass consists of about 45%–60% proteins, 10%–15% lipids and 10%–15% nucleic acids (measurements on Chinese hamster oocyte cells under different conditions shown in Fig. 3A). Accordingly, different physical principles have been used for measuring the cell density. As protein accounts for the majority of the dry mass of a cell, protein concentration is often used as a surrogate for relative cell density. Relative protein concentration can be measured using primary amine-reactive fluorescent dyes, such as N-hydroxysuccinimide ester, which forms covalent bonds with all proteins in a cell (Kafri* et al.* 2013). Thus the fluorescent intensity is proportional to the protein concentration. Although membrane permeable versions of such dyes are available, temporal tracing of protein concentration in living cells is not feasible, since it would require quantitatively invariant staining over and over to the same cells.

The second type of method is Raman imaging. Lipid and protein molecules have different characteristic peaks in Raman spectra (Fig. 3B). These characteristic peaks can be used to quantify the concentration distribution of lipids and proteins in the cell (Fu* et al.* 2012). Raman imaging measures the sample’s Raman spectra point by point, which is then used to generate spatial maps of protein and lipid concentration. The sum of these two concentration distributions is approximated as the cell density distribution. Measuring the concentrations of nuclei acids and polysaccharides is also possible if more Raman bands are quantified (Oh* et al.* 2019). Noteworthy, size information can be extracted from the lipid distribution, since the lipid membrane appears as clear boundaries in these lipid maps. Under similar principles, this method can also be used for characterizing subcellular structures with distinct density composition, such as oil-enriched compartments (*e*.*g*., lipid droplets in hepatocytes) or protein aggregation (*e*.*g*., amyloids in Alzheimer model mouse brain) (Oh* et al.* 2019). However, the Raman imaging is a scanning-based method, with limitations on imaging speed. Implementation of this method requires a sophisticated optical setup to eliminate auto-fluorescence from cells and to reproducibly measure the stimulated Raman scattering light (Fu* et al.* 2012; Oh* et al.* 2019).

The third type of method uses the principle that the optical refractive index *n* of a cell linearly correlates with protein and DNA concentration as shown in Fig. 3C, and the linear factor α (α = d*n*/d*c*) is roughly 1.8 × 10^–^^4^ m^3^/kg (Barer 1952; Mir* et al.* 2011; Zangle and Teitell 2014). The d*n*/d*c* value of other components such as sugars, dipalmitoyl phosphatidylcholine and NaCl ranges from 1.4 × 10^–4^ to 2.2 × 10^–^^4^ m^3^/kg at physiology concentration (Zangle and Teitell 2014), which is close to the α value. Thus, the cell density ρ can be estimated from the optical refractive index *n* and this linear factor α. The refractive index *n* of a single cell can be calculated from the spatial phase of the light. As a simple example in Fig. 3, when a plane wave passes through a cell with thickness *d*, its optical path (defined as *n* multiplying the distance that light travels) differs from the one passing the area without cells. This retardation of light causes a phase shift *ϕ* = (*n* – *n*_0_)*d*. From the phase shift information, one can derive the cell geometry and refractive index distribution. It should be emphasized that the phase information from only one light-axis is not sufficient for reconstructing the 3D density distribution, similar to the fact that reconstructing a 3D object from one projection is impossible. However, the cell mass can still be calculated under this situation. The cell mass *m* can be calculated as follows:

$ m = \iiint {\rho {\text{d}}x{\text{d}}y{\text{d}}z = }\iint {\left(\int\limits_{{{\text{z}}_1}}^{{{\text{z}}_2}} \rho {\text{d}}z\right) }{\text{d}}x{\text{d}}y = \frac{1}{\alpha }\int {\phi \lambda {\text{d}}A} , $

where λ is the light wavelength, *A* is the cell projection area,* z*_1_ and *z*_2_ are the *z* coordinate of the bottom and top of the cell membrane at the location of (*x*, *y*) respectively. In this situation, $\displaystyle \int\limits_{{z_1}}^{{z_2}} {\rho {\text{d}}z = \frac{{\phi \lambda }}{\alpha }} $ can be viewed as “2D density” distribution ρ_s_.

There are different ways to measure the phase shift: digital holography (Cuche* et al.* 1999), wavefront sensing camera (Liu* et al.* 2020), interferometry microscopy (Reed* et al.* 2011), and Fourier ptychographic microscopy (Ou* et al.* 2013). To get the 3D refractive index distribution, one needs to acquire the quantitative phase shift information from multiple directions by either changing the incident angle of the coherent light (Shin* et al.* 2015, 2018) or rotating the sample (Charrière* et al.* 2006a, b). For a more comprehensive introduction of quantitative phase imaging we recommend another recent review (Park* et al.* 2018).

The relative density of a cell can also be derived from its crowding state (Neurohr* et al.* 2019). Molecular crowding at different size scales can be directly measured with the diffusion of fluorescent particles, either synthetic (Daniels* et al.* 2006) or genetically encoded (Delarue* et al.* 2018). This method is amenable to microscopy and therefore allows recurrent measurement of single cells over time. Nevertheless, while diffusion can reflect the change of cell density, it can also be influenced by other physical properties. Thus, the conclusions must be made with careful controls and interpretations.

## CONCLUSIONS AND OUTLOOK

Living cells are dynamic agents that process information and energy from the environment to sustain their internal activities such as growth and division. While structure determines function, in living systems, the structure is also actively maintained or altered by the function. The interaction between cell size and cell cycle is such an example: cell cycle can be regulated by the cell size via a size checkpoint (Johnston* et al.* 1977); conversely, cell growth (increase in size) halts when cell cycle progression is blocked (Ginzberg* et al.* 2018). In such a system that is inherently nonlinear and full of feedbacks, it is with the dynamical information of cell size and cell state can we begin to understand the dialectic relationship between size and function. While many experimental methods described above allow recurrent measurement of the same cell, some only capture snapshots of size and growth parameters. Computation methods, including the ones developed in other single-cell fields, may be used to infer temporal dynamics from fixed-cell data (Kafri* et al.* 2013). In addition to developing more precise and accessible methods to measure single-cell size and growth, combining them with other single-cell methods, such as single-cell omics and time-lapse microscopy, holds great promise to answer some of the most fundamental questions of cell size control. How do cells sense their size? How is cell size coupled to cell fate decision and lineage specification? How do cells regulate their size in the cell cycle, or do they use the cell cycle to regulate their size? How do cells lose size homeostasis in disease and how does this contribute to the development of disease? The quantitative dynamics of cell size, cell state and cell signaling in single cells are essential in answering these questions. With rapidly advancing technologies, exciting times lie ahead.

## Conflict of interest

An Gong and Mingwei Min declare that they have no conflict of interest.
